# Current Research Status and Future Trends of Vibration Energy Harvesters

**DOI:** 10.3390/mi15091109

**Published:** 2024-08-30

**Authors:** Guohao Qu, Hui Xia, Quanwei Liang, Yunping Liu, Shilin Ming, Junke Zhao, Yushu Xia, Jianbo Wu

**Affiliations:** 1 School of Mechanical Engineering, Sichuan University, Chengdu 610065, China; quguohao@stu.scu.edu.cn (G.Q.); xh@scu.edu.cn (H.X.); wujianbo@scu.edu.cn (J.W.); 2 Dongfang Electric Group Dongfang Electric Motor Co., Ltd., Deyang 618000, China; liuyunping@dongfang.com (Y.L.); mingshilin@foxmail.com (S.M.); zhaojk@dongfang.com (J.Z.); xiays0795@dongfang.com (Y.X.)

**Keywords:** self-powered, vibrational energy, piezoelectric switching, electromagnetic switching, friction electric switching, electrostatic switching, magnetostrictive switching

## Abstract

The continuous worsening of the natural surroundings requires accelerating the exploration of green energy technology. Utilising ambient vibration to power electronic equipment constitutes an important measure to address the power crisis. Vibration power is widely dispersed in the surroundings, such as mechanical vibration, acoustic vibration, wind vibration, and water wave vibration. Collecting vibration energy is one of the research hotspots in the field of energy. Meanwhile, it is also an important way to solve the energy crisis. This paper illustrates the working principles and recent research progress of five known methods of vibrational energy harvesting, namely, electromagnetic, piezoelectric, friction electric, electrostatic, and magnetostrictive vibrational energy harvesters. The strengths and weaknesses of each method are summarised. At the end of the article, the future trends of micro-nano vibrational energy collectors are envisioned.

## 1. Introduction

Along with the advancement of the global economy, the industrial development of various countries has been rapid, and along with it, the industrial system has also been gradually improved. On the one hand, in industrial production, the use of large equipment of all kinds is usually accompanied by a lot of energy wastage, like heat and mechanical energy, which can lead to equipment damage and reduce the life of the equipment and productivity, so it is particularly important to monitor the health of the equipment. On the other hand, the development of IoT technology has been associated with the use of a great deal of miniature sensors with ultra-small dimensions and high-precision measurements, low power consumption and intelligence, etc., which are now widely used in medicine, environmental monitoring [1], security, and other fields [2].

The applications of miniature sensors to collect parameters during the operation of equipment for health monitoring is the most used method today. However, these sensors usually require continuous operation, and therefore, energy supply becomes a major problem for these sensors to work properly over an extended period of time. Using commonly traditional chemical batteries is limited by their limited life span, chemical contamination, large mass, large size, and other defects. Especially in human implantable microdevices [3], it is difficult to apply conventional chemical batteries. As a result, researchers at both the national and international level are working on ways to recover energy from the surroundings to achieve self-sufficiency in electronic devices, and the rapid advances in micron and nanotechnology, mechanical, and bill-of-materials projects have made it possible to harvest vibrational energy from the environment [4]. These vibration energies not only come from industrial equipment but also come from the vibration of vehicles [5], sound vibration, water vibration, and even the vibration of the human heart, etc., which are very clean and widely sourced. At present, harnessing vibrational energy in the environment for self-powering has become a popular research project. Collecting vibration energy in the environment has a wide variety of applications; in addition to realising the self-powering [6] of miniature sensors, it is also of great significance for energy recycling and improving energy utilisation. In addition, quantitative harvesting of vibrational energy has become a major research interest, and by quantifying the resonance wavelength dependence of vibrational energy in plasma-based molecular systems, it can contribute to future applications of tailor-made systems with controlled energy transfer pathways [7]. However, the amplitude of vibration sources in the surroundings is often small, and the rate of vibration varies considerably from one source to another. This requires vibration energy harvesters with broadband characteristics in the low amplitude range [8].

Currently, scholars both domestically and internationally have proposed a variety of vibration energy collectors, mainly electromagnetic vibration energy collectors [9], piezoelectric vibration energy collectors, friction electric vibration energy collectors, electrostatic vibration energy collectors, and magnetostrictive vibration energy collectors. In this paper, we review the basic principles of these vibratory energy harvesters, then compare the strength and weaknesses of these energy collectors, and systematically introduce the current problems of vibratory energy harvesters to look forward to the future development trend.

The remainder of the article is organised as follows. Section 2, Section 3, Section 4 cover the working principles and research status of electromagnetic, piezoelectric, friction electric, electrostatic, and magnetostrictive vibration energy harvesters, and Section 7 summarises the various forms of vibration energy harvesters in the preceding sections and describes the views on vibration energy harvesting technology.

## 2. Electromagnetic Vibration Energy Harvester

### 2.1. Working Principle and Characteristics of the Electromagnetic Vibration Energy Harvester

According to Faraday’s law of electromagnetic induction, the electromagnetic vibration energy collector is designed to convert external random mechanical vibrations into movements of a coil ring or permanent magnet, achieving relative motion between the two, which causes a shift in the magnetic permeability flux in the coil ring, producing induced electromotive potential. Based on the vibration content, electromagnetic vibration energy collectors based on electromagnetic conversion can be classified into three types: moving iron, moving coil, and iron coil with vibration [10]. Depending on the structure, electromagnetic vibration energy harvesting (EMVEH) could be divided further into linear electromagnetic vibration power capture and rotational electromagnetic vibration power harvesting. Linear electromagnetic vibration energy harvesting (EMVEH) mainly consists of two components: coil and permanent magnet. The principle of operation is the generation of a recurrent linearity vibration that excites a contrasting linear motion between the loop and the magnet, thus producing an electromagnetically induced current in the loop. The revolving EMVEH consists mostly of a dynamo and a mechanical movement rectifier mechanism for rectifying the exterior vibrations [11].

**Figure 1:**
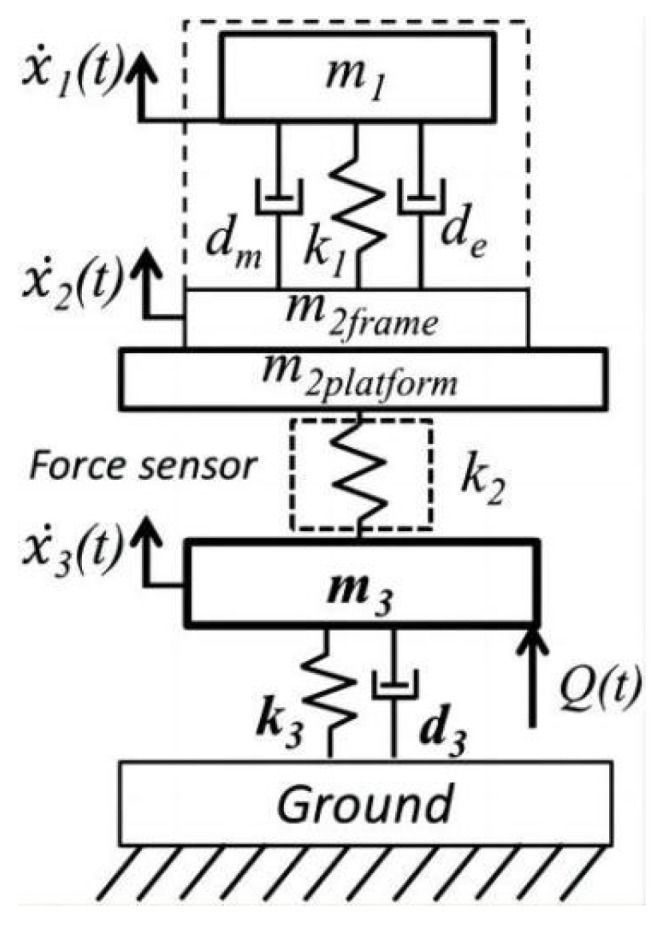
Linear mechanical system with energy harvesting system for vibrating structures [12].

Vibration energy harvesters must resonate at a certain frequency and level of vibration to maximise energy output; however, the frequency and level of vibration in the environment is often variable and not quite suited to sustained resonance, thus having a significant impact on the performance of vibration power capture. Zdenek et al. presented a linear mechanical mode of a vibratory energy harvesting system (see Figure 1) [12], which consists of a vibrating structure, a vibration source, a force transducer, a harvester frame, and a vibratory energy collection system. In addition, a genetic model of the electromagnetic translators was included in the mechanical model to calculate the dissipative feedback and to provide electromagnetic dampers for the energy harvesting mass springs on the energy harvesting system. The phantom was used to measure the interior mechanical stress F between the frame of the vibration energy collector and the vibrating construction. The efficiency was then solved using Equation (1), i.e.,

(1) $$\eta =\frac{E_{electric}}{E_{mech}}=\frac{\int_{t_{1}}^{t_{2}}u(t)\cdot i(t)dt}{\int_{t_{1}}^{t_{2}}F(t)\cdot {\dot{x}}_{2}(t)dt}$$

Scrutinising the export voltage and current of the circuit, the current and voltage can be obtained, and the speed of the mechanical vibration can be measured with an ordinary vibrometer [12].

The vibrating structure is a mass-spring-damping system with one degree of freedom (m_3_, k_3_, d_3_) containing the mechanical part of the vibrating energy harvester (m_1_, k_1_, d_m_, d_e_), whose resonance value can be adjusted to the intrinsic value of the vibrating structure. The force transducer is modelled as a mass-spring system (m_2platform_, k_2_), which allows for fixing the framework of the power collector to the vibrating frame. The mechanical force information of the vibrating energy harvester is calculated from the value of k_2_ of the spring in the force sensor and then from Equation (2).

(2) $$F=k_{2}\left(x_{2}-x_{3}\right)$$

### 2.2. Advances in Electromagnetic Vibration Energy Harvesters

**Figure 2:**
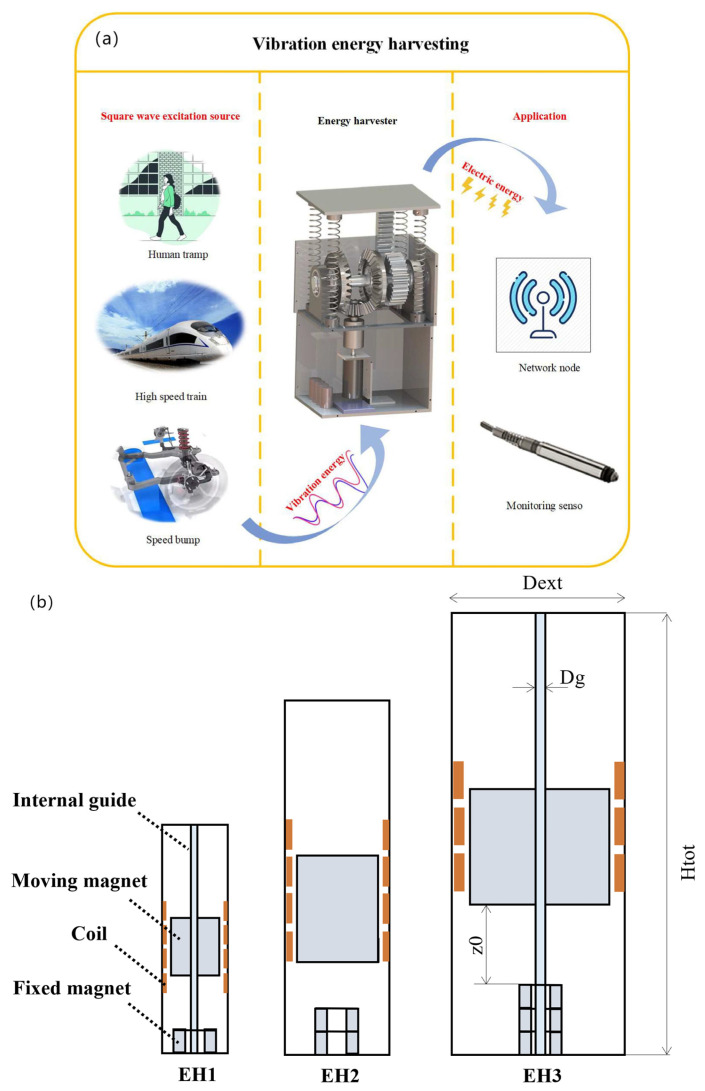
(**a**) Vibration-energy-collecting devices for speed traps, pedestrians, and high-speed trains [14]. (**b**) Schematic structure of a gravity electromagnetic energy harvester based on magnetic levitation fluid [15].

The electromagnetic vibration energy harvester has a simple structure and large output current, and it now is a relatively mature technology, emergent in commercial products. However, scientists are still working hard to improve the energy transformation efficiency and power intensity. Cui et al. [13] carried out a mathematical and experimental study to analyse the behaviour of the devised energy sensor’s response in a low-acceleration environment using a unique combination of a basic equivalent circuit mode and a magnetic farm simulator. The research team composed the energy sensor using four electromagnets, two I-shape cores and two U-shape cores composed of light ferrite material, and a coil for inducing voltage. The dimensions of this electromagnetic energy sensor are 43 × 37 × 80 mm^3^. Based on the theoretical modelling of the sensor, the mean MAX power of the energy sensor is 3.24 mW at a rate of 28 Hz with an acceleration of 0.4 g, and 3.83 mW at a rate of 31 Hz and an acceleration of 0.5 g [13]. To make this vibration energy harvester have a wider range of applications, such as applications in speed limit vibration, railway rail vibration, and body stepping conditions, Wang et al. presented a vibration energy harvester (VEH) system built on a gear rack mechanism and a bevel gear set (e.g., Figure 2a) [14]. While the power collector is deployed in an area subjected to a square wave inhibition, for example, from deceleration zones or fast-moving trains, the movement conversion device transforms the vertical oscillating movement of the entry plate and rack caused by the outside force to a bi-directional spinning of the spur gears. Subsequently, the rectification unit changes the bi-directional revolution of the spurred gears to a one-way revolution of the conical gears. Finally, the one-way movement of the conical gears drives the alternator, which generates electrical energy. The power storage unit then accumulates the rectified and voltage-enhanced electrical energy in a supercapacitor, which powers low-energy sensors. The system has a maximum power of 2.7 W, a 66% increase in maximised power in contrast to sine excitation with the same conditions [14].

In addition, Peng et al. studied the influence by wire diameter of the power exported from an electromagnetic energy collector, and the experimental results showed that the optimal wire diameter produces a MAX mean power of 37.45 mW at 20 Hz and 1.0 g excitation [15]. Monaco et al. devised a procedure for the description and optimisation as a gravity-electromagnetic energy collector (GVEH) on the basis of a magnetically levitated fluid (MLF) (Figure 2b) [16]. The system consists of a movable magnet in suspension, a stationary magnet at the foot, and a coil winding around the tube. There are three configurations, which depend mainly on the axial position of the magnet, the dimensions of the tube, the internal guides, and other components, with the three configurations being EH1, EH2, and EH3. The system has an external excitation with an amplitude of 0.5 g, producing a MAX RMS power of 32 mW at a resonance frequency of 4 Hz [16]. Sun et al. provided an optimal coil design by traversal calculation to maximise the MAX export power and MAX power density [17]. The total volume of the design (EVEH) is 7.6 cm^3^. At a vibration acceleration of 1 g and a vibration rate of 116 Hz, the EVEH has a MAX output power of 27.2 mW, giving a power density of 3.6 mW/cm^3^ [17]. Zakaria et al. investigated the EH behaviour of a retarded self-excited oscillator coupled to a retarded electromagnetic collector and showed that there are frequency optima for the maximum QP amplitude and output power in the absence of a delay in the electromagnetic coupling [18]. By adjusting the delayed amplitude in the circuit, the energy collector can be kept in a slow-amplitude oscillatory state, thus improving the power output performance. Lorenzo et al. presented an adjustable multi-armed electromagnetic pendulum for energy capturing from ultra-low-frequency vibrations and enhanced the initial configuration, which comprises five solenoid sensors and a magnetic coil magnet, each of which is supported by a swing arm of varying lengths: this six-armed framework is freely oscillatable around a central pivot when stimulated by external vibrations [19]. Experimental findings indicate that the proposed energy harvester is competent to operate within the extremely low frequency band around 2 Hz to 10 Hz. The design of a multiple eccentric coil electromagnetic leapfrog energy collector (MEGEH) was presented by Xiong et al. [20]. The effects of the placement and number of sensing coils on the export properties of the energy collector were investigated. The experimental findings indicated that a higher number of coils improves the power collector, but the ratio of the improvement of the output power would be reduced. The total output power levels are 0.4 mW, 0.62 mW, and 0.72 mW [20].

The development of electromagnetic vibration power collector technology is now comparatively mature. Electromagnetic vibration energy harvesters are able to collect vibration energy at low, medium, and high frequencies, but the problems of higher cost and limited output power are still a major challenge for electromagnetic vibration energy harvesters. Table 1 lists the experimental data of some electromagnetic vibration energy harvesters for a more intuitive comparison.

## 3. Piezoelectric Vibration Energy Harvester

### 3.1. Operating Principle and Characteristics of Piezoelectric Vibration Energy Harvester

The piezoelectric vibration energy harvester consists of several piezoelectric generators integrated together; the fundamental principle of the piezoelectric generators is the piezoelectric effect [8]. When deformed in a certain direction by an external force, its internal polarisation phenomenon will occur, and at the same time in the two opposite surfaces of the positive and negative charges. Whether the external load is withdrawn, it will be restored to the uncharged state—this phenomenon is known as the positive piezoelectric effect. When the external force changes to another direction, the polarity of the charge also varies. Conversely, while the electric force acts along the polarisation direction of the force in the dielectric, these dielectrics are also deformed, and the deformation of the dielectrics disappears when the electric field is withdrawn, a phenomenon referred to as the inverse piezoelectric interaction.

**Figure 3:**
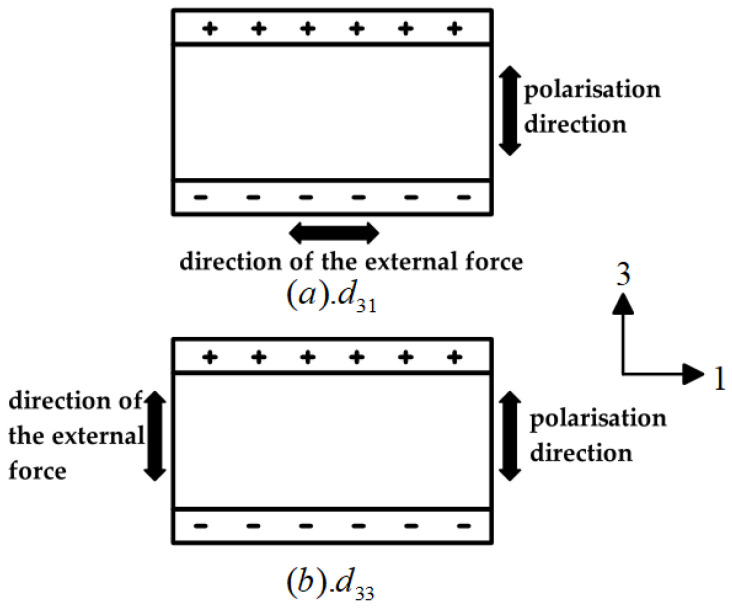
(**a**) d_31_ operating mode. (**b**) d_33_ operating mode.

During the process of vibration energy capturing via piezoelectric elements, the positive piezoelectric effect is mainly utilised to realise the transformation of mechanical vibration to electrical power. In practice, the energy conversion efficiency of piezoelectric materials is as high as possible; thus, it is indispensable to take into account the size, shape, and type of piezoelectric materials, in addition to its mode of operation. When the external input excitation size and phase are the same, the output of piezoelectric materials with different operating modes will be significantly different [22]. Within the area of piezoelectric vibration energy collection, two common work modes for piezoelectric materials are d_31_ and d_33_. dij is a piezoelectric constant that is used to characterise piezoelectric materials [23]. The first cursor i shows the direction of polarisation and the second cursor j shows the orientation of the external force. The working modes of piezoelectric materials are shown schematically in Figure 3.

As can be seen from Figure 3, the d_31_ working mode has a stress direction of 1 and a polarisation direction of 3, which are orthogonal to each other; the d_33_ working mode has a stress direction of 3 and a polarisation direction of 3, which are parallel to each other. The difference between the above two modes is not only that the stress direction is different from the polarisation direction, but also that the piezoelectric constants of the two modes are quite different. In terms of the voltage and charge outputs in these two modes of operation, when the circuit is open, they can be expressed as follows [24]:

(3) $$d_{31}:\;\left\{\begin{array}{c} V_{31}=\sigma g_{31}H\\ Q_{31}=-\sigma A_{elec(31)}\;d_{31} \end{array}\right.$$

(4) $$d_{33}:\;\left\{\begin{array}{c} V_{33}=\sigma g_{33}H\\ Q_{33}=-\sigma A_{elec(33)}\;d_{33} \end{array}\right.$$

where Q is the amount of charge generated (in coulombs, C), d is the piezoelectric coefficient of the piezoelectric material (in coulombs/Newtons, C/N), A is the area of the force exerted on the piezoelectric material (in square metres, m^2^), σ is the stress (in Newtons, N), g is the dielectric voltage factor (in V_m_/N or m^2^/C), H is the thickness of the piezoelectric stratum (in metres, m), and the relationship between g and d can be expressed as follows:

(5) $$g=\frac{d}{\varepsilon }$$

where ε is the dielectric constant.

Materials with positive and negative piezoelectric effects are referred to as piezoelectric materials. Piezoelectric materials are extensively utilised in the fields of optoelectronics, mechanics, and electronics, etc., due to their precise response, ease of use, and high energy density [25]. To quantify the properties of piezoelectric materials, piezoelectric coefficients and electromechanical coupling coefficients were introduced to describe them. Piezoelectric coefficients are characterised for the energy conversion efficiency of piezoelectric materials—the higher the piezoelectric index, the higher the efficiency of energy conversion. When the mechanical force is imposed, due to the piezoelectric phenomenon, a portion of the mechanical energy should be transferred to electrical power, and the intensity is characterised by the electromechanical coupling coefficients, which is a dimensionless quantity [23]. Piezoelectric materials commonly available for vibration energy capture include PZT (lead zirconate titanate), AlN, ZnO, and PVDF (polyvinylidene fluoride) [24]. The electromechanical coupling coefficients and piezoelectric constants of common piezoelectric materials are shown in Table 2.

### 3.2. Progress of Research on Piezoelectric Vibration Energy Harvesters

**Figure 4:**
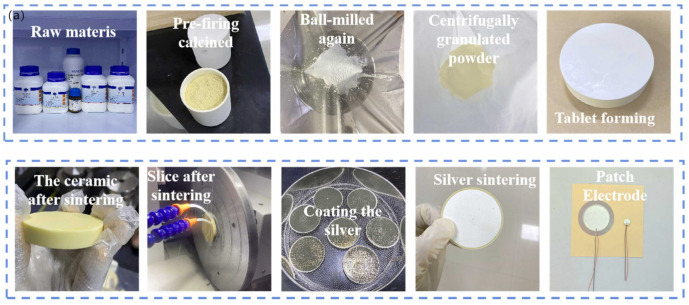

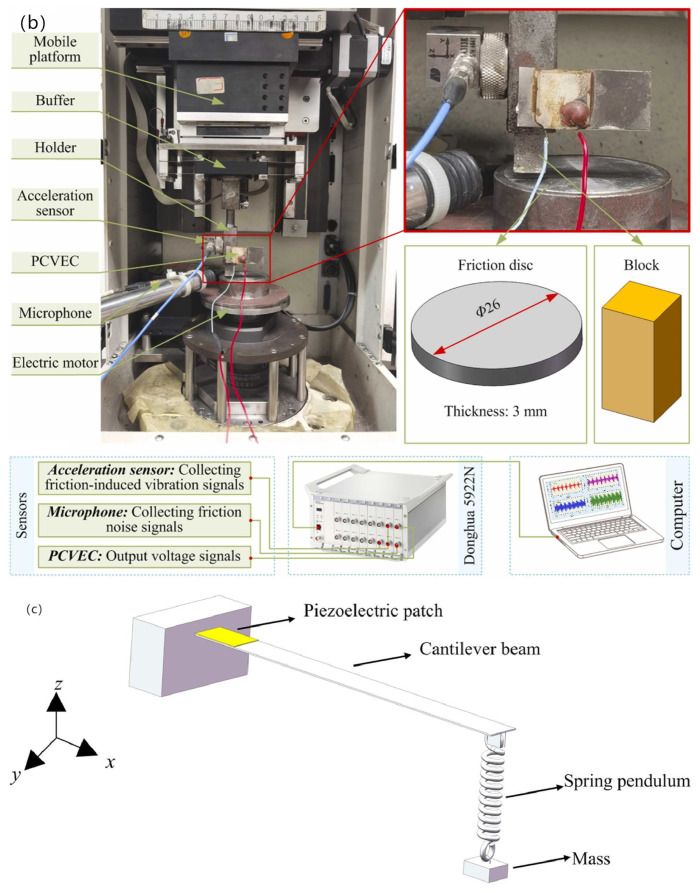

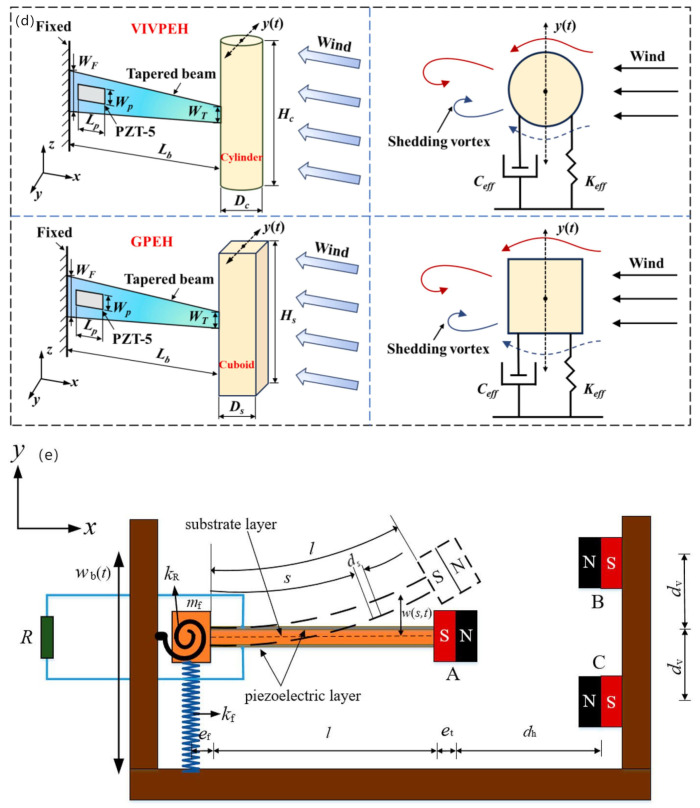
(**a**) Physical diagram of PSN-PZT piezoelectric ceramic product [28]. (**b**) Friction vibration test rig [29]. (**c**) Cantilever beam-spring-pendulum structure [30]. (**d**) Schematic diagram of conical beam FIVPHEH [31]. (**e**) Configuration diagram of the TPEH + DM system [32].

Piezoelectric vibration energy capture is a major technology for vibration energy capturing, with the benefits of high power density and energy density, and it has been applied in the following areas: wearable electronic devices and biomedical devices, etc. As technology keeps advancing, piezoelectric materials will be enhanced. In terms of energy harvesting technology for road vibration relying on piezoelectric effects, as early as 2006, Fang et al. [26] presented a miniature cantilever beam piezoelectric vibration energy collector, which uses a complex cantilever with a 1.64 μm PZT level to collect the vibration energy, the overall cantilever length × width of 2000 × 600 μm^2^, and the length × height of the Ni-metal mass of 600 × 500 μm^2^, with the experimental finding indicating that at a resonance value of 609 Hz and an accompanied acceleration of 1 g, the voltage output value is 898 mV and the output power is 2.16 μW [26]. In 2008, Shen et al. [27] developed a microelectromechanical system (MEMS) piezoelectric-energy-collecting device, which is a single-crystal PZT cantilever with an embedded anti-silicon mass block, for low-vibration-rate and high-vibration-magnitude environments. The results demonstrated that the device was capable of generating an export peak–peak voltage of 160 mV_pk_ and an export power of 2.15 μW, with an energy intensity of 3272 μW/cm^−3^ at 461.15 Hz, 2 g acceleration, and 6 kΩ [27]. In 2022, Ye et al. developed a PSN-PZT phenocrystal solution for road vibration power collection system, and Figure 4a provides the procedure for the formulation of PSN-PZT piezoelectric ceramics, which consisted of zirconia-lead titanate PZT piezoelectric cermets prepared by the equal and non-equal mixing of A-site B-lead ions with different polarisation parameters [28]. PSN-PZT piezoelectric ceramics are fabricated through conventional ceramic sintering. Firstly, the metal oxide powder is dried and weighed. Subsequently, it is ground in a mill, during which zirconium balls and deionised water are added. The slurry is dried at 1000 °C and pre-sintered. The pre-sintered powder is re-ball milled with 10% polyvinyl alcohol binder, zirconium balls, and deionised water. After ball-milling, it is processed by centrifugal spray granulation and drying mechanisms into a flowable powder. The preparation is compressed and placed in a chamber degreasing oven for 30 min, then sintered at 1300 °C for 3 h. After sintering, it is sliced into 0.2 mm sheets with a wire cutter and then ground smoothly by a double-sided grinder, coated with electronically conductive silver paste, and then placed in the box-type silver firing furnace for 20 min. Lastly, the piezoelectric cells are placed in hi-heat silicone oil to obtain piezoelectric ceramics under different polarisation conditions. Piezoelectric certificates with piezoelectric characteristics are then bonded to 304 stainless steel using a structural adhesive to create a drum-type piezoelectric certificates power conversion device, which can be obtained by curing the PSN-PZT piezoelectric certificates in an electrically heated blast drying oven. The optimum polarisation conditions were obtained by analysing their respective performances as 70, 15 min, and 3.75 kV/mm. Based on these properties, the dielectric behaviour is characterised by the piezoelectric constant d_33_ = 857 PC/N, the electromechanical coupling coefficient K_p_ = 0.783, the mechanical quality factor Q_m_ = 38.67, and the Curie temperature T_c_ = 184 °C, which ensures the stability and security of the road piezoelectric sensors. The tests indicate that the piezoelectric sensor unit of PSN-PZT has an export voltage of 52.89 V and an export power of 35.01 mW (10 Hz) at an excitation displacement of 2 mm [8]. Xiang et al. developed a piezoelectric cantilever vibration power collector to collect the friction-induced vibration power (friction vibration test rig is shown in Figure 4b) [29]. A sample of the friction block assay is attached to the clamps mounted on the motor. The FIV and Noise (FIVN) characteristic is simulated by applying a vertical force on a two-dimensional moving platform to ensure that the block is in contact with a spinning friction plate. The FIV power produced by the gliding friction interaction among the rubbing module and the rubbing discs can be efficiently converted into electrical energy. The results show that improving the FIV intensity on the friction surface of the PCVEC friction interface and the stimulus source can significantly increase the output voltage; when the quality and the rigidity of the cantilever beam are low, the PCVEC exhibits higher deformation capacity, which enables the piezoelectric ceramic model to generate more vibration energy than the friction block, converting FIV energy into electrical energy [30]. To increase the collection efficiency of piezoelectric vibration energy harvesters, Zhang et al. provided a multidirectional energy collector with a cantilever beam-spring swing design (Figure 4c) [30], where x and y are directed toward the length and width of the cantilever beam, and z is along the direction of gravity. The pendulum is suspended in the z-orientation, and the flexural motion of a bridge twinned with the nonlinear motion of a swinging pendulum in two directions, which is capable of responding to multidirectional vibrations and depends on the low fundamental frequency of the swing. At low frequencies, the function-enhanced piezoelectric girder-spring pendulum device produces 106.6% and 187.3% of the maximum output voltage amperes of a piston girder-single pendulum under x- and z-direction excitations, respectively [30]. Wang et al. found that for commonly used cantilever beam piezoelectrics, the form of the cantilever beam affects the stress profile on the face, thus leading to variations in the piezoelectric strain [31]. In contrast to other cantilever beams, the stress distribution of conical beams is more uniform. Therefore, the research group proposed a conical camel-beam flow-induced vibrational piezoelectric energy collector, combining a conical beam with a flow-induced vibrational piezoelectric energy harvester (FIVPEH) with a total size of 196 mm × 32 mm × 120 mm. The principle is depicted in Figure 4d [31]. The conical beam piezoelectric harvester (PEH) comprises a supporting structure; a conical camel beam; and a phenolic film, the PZT-5. The camber beam is made of virgin aluminium, and the support structure is made of Styrofoam. The supporting structure and the piezo film are fixed to the top and fixed end of the camel beam, respectively. The camber beam is fastened onto an aluminium framework. The results of the experiments show that the strain distribution of the conical beams is more homogeneous, has greater equivalent rigidity, and increases the width of the stationary end, enhancing the output voltage and reducing the vibrational dispersion. The MAX voltages of the conical beams VIVPEH and GPEH were 19.82 V and 21.00 V, which were higher than those of the rectangular beams [31]. A triple-stable piezoelectric energy collector with an integrated dynamic magnifier (TPEH + DM) was proposed by Man et al. (e.g., Figure 4e) [32]. The system utilises a transverse and rotating spring-mass-based system to amplify the vibration amplitude of a cantilevered piezoelectric beam, thereby effectively harvesting the energy of the low-orbit vibrations [32]. The energy-harvesting device comprises a double cantilever piezo beam and two external magnets mounted on a U-block. Furthermore, a digital amplifier is put among the stationary end of the piezo beam and the U-block. The mass of the fixed end of the system is denoted by M_f_; the mass of the tip magnet is denoted by M_t_; and the vertical and rotary springs are represented by k_f_ and k_r_, respectively, with two external magnets, labelled A and B, are mounted symmetrically on the right side of the U-block. The cantilevered piezoelectric assembly has a total length l and consists of a metallic coating of thickness h_s_ and a couple of dielectric layers of thickness t_p_, which are attached to their upper and lower sides, respectively. The distances horizontally and perpendicular to each other across the tip magnet and the outside magnet are denoted as d_h_ and d_v_, respectively, thereafter. The mass eccentricity of the fixed end of the beam and the tip magnet are denoted as e_f_ and e_t_, respectively.

In general, the piezoelectric vibration energy collector features characteristics such as high energy density and simple structure. The main influencing factor of the piezoelectric vibration energy collector to collect energy still lies in the piezoelectric material, and a variety of high-voltage electric ratios and high mechatronics coupling coefficients of the piezoelectric material have been developed, but the future research in this area is still a big hot spot. And the piezoelectric material having low strength, mechanical coupling, easy aging, and other problems is still a big challenge. Table 3 lists the experimental data of some piezoelectric vibration energy harvesters for a more intuitive comparison.

## 4. Friction Electric Vibration Energy Harvester

### 4.1. Mechanism of Operation and Characteristics of the Friction Electric Vibration Energy Harvesters

#### 4.1.1. Friction Nanogenerator

The friction nanogenerator (TENG) was first developed by American scientist Zhonglin Wang as a micro-generator that can rely on the charge-pumping effect of the electric potential at friction points to convert extremely small amounts of mechanical energy into electrical energy. Electrocution by friction is among the most common occurrences in nature, no matter if combing hair, getting dressed, walking, or driving a car. However, friction electricity is difficult to collect and utilise, so it is often overlooked. A research group led by Georgia Tech professor Zhonglin Wang has developed a transparent flexible friction nanogenerator that successfully converts friction into usable electricity with the help of a flexible polymer material [37].

Friction nanogenerators rely on the charge pumping effect of the friction point potential to produce electricity by friction between polyester fibre sheets and polydimethylsiloxane (PDMS) sheets [38]. With the help of detachment technology, when friction occurs, a charge separation and potential difference is created between the two polymer films, which can be used to generate an electric current through an outside circuit. During friction, the polyester fibres produce electrons, while the polydimethylsiloxane receives them. In addition, mechanical deformation from external pressure causes them to frictionally generate electricity [37]. In addition, the vibration energy in the surroundings is also the reason for this mechanical deformation, so using vibration power in the surroundings to provide a source of energy for the friction nanogenerator is one of the main directions of TENG’s research nowadays.

#### 4.1.2. Principle of Friction Electric Vibration Energy Harvester

**Figure 5:**
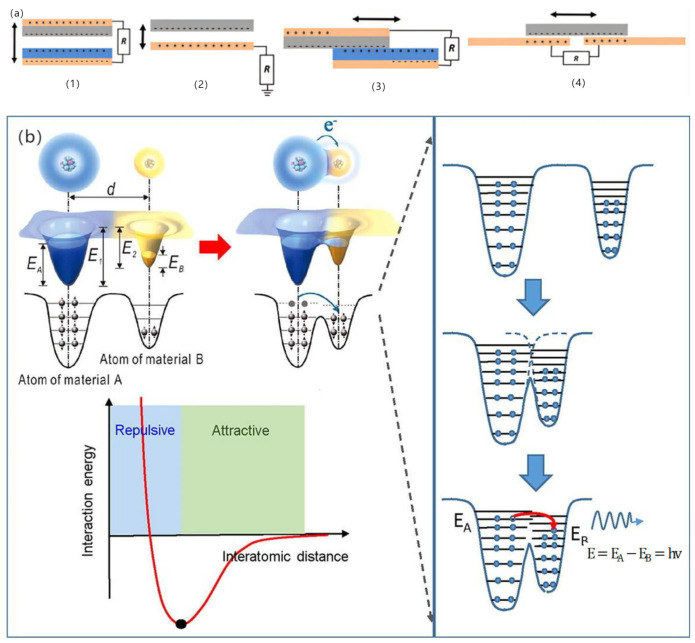
(**a**) Four modes of friction nanogenerators [39]. (**b**) Potential well model of electron cloud [40].

A friction electric vibration energy harvester generally consists of several friction nanogenerators, which is done to increase the energy harvesting efficiency. Friction nanogenerators (TENG) have four paradigms of operation: vertical access separation pattern, in-plane sliding pattern, single-electrode pattern, and independent friction electric layer pattern (see Figure 5a below) [39]. In the vertical contact separation configuration, two surfaces made from distinct materials come into contact and are subsequently pulled apart in a vertical direction. This interaction leads to the generation of opposite electrostatic charges on the surfaces. In the single-electrode configuration, triboelectric charging occurs through contact and separation, similar to the contact separation configuration, but utilises the ground as the reference electrode. The lateral sliding configuration shares the common architecture and initial location with the vertical touch release configuration; however, the triboelectric charge is produced by sliding the two surfaces in a relatively parallel manner. The fundamental concept of the freestanding friction layer configuration involves connecting a symmetrical pair of poles to an outside circuit, allowing the movement of the freestanding friction layer between them to enable current flow from one electrode to the other. According to the electron cloud potential well model by academician Zhonglin Wang, the essence of friction electrification is the transfer of contact charge on the surface of a material. Matter is composed of atoms, and when the distance between two atoms attributed to different materials is less than the equilibrium distance (or bond length), the electron clouds overlap, and the charge transfer occurs, and in this way, an electric current is formed [40] (see Figure 5b for details).

The principle of the friction nanogenerator can be well explained in terms of Max-well’s displacement current: see, for example, Equation (6):

(6) $$J_{D}=\frac{\partial D}{\partial t}=\varepsilon_{0}\frac{\partial E}{\partial t}+\frac{\partial P}{\partial t}$$

where J_D_ refers to shift flow; D refers to shift field; E refers to electric scene; P refers to polarisation scene density; and ε_0_ refers to the vacuum dielectric constant. The first term in Equation (6). $\varepsilon_{0}\frac{\partial E}{\partial t}$ represents the generation of electromagnetic waves in the displacement current, which was later used as a method for the development of radio, radar, television, and long-distance wire-free access to communication. The second item $\frac{\partial P}{\partial t}$ shows the correlation from the displacement current to the export signal of the nanogenerator.

### 4.2. Advances in Friction Electric Vibration Energy Harvesters

**Figure 6:**
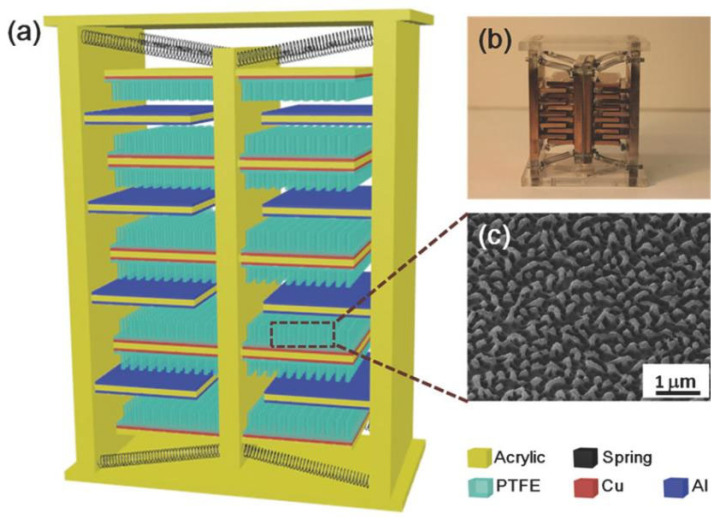
(**a**) Schematic diagram of 3D-TENG; (**b**) SEM image of nanopore on an aluminium electrode; (**c**) photograph of the fabricated 3D-TENG [41].

**Figure 7:**
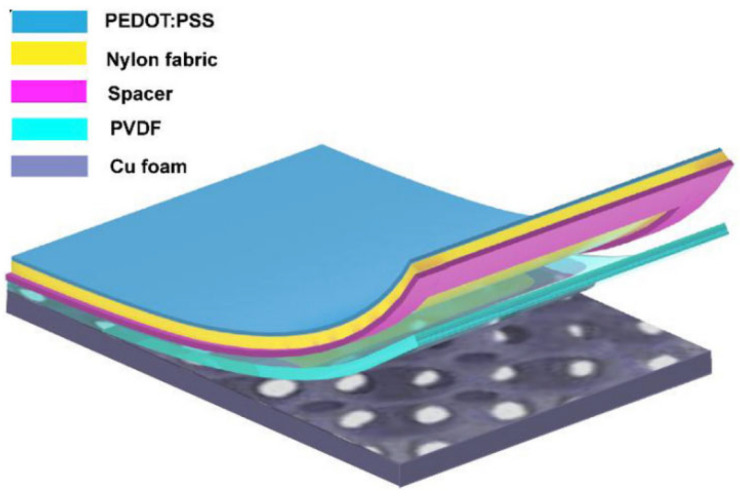
Structure of TENG [42].

**Figure 8:**
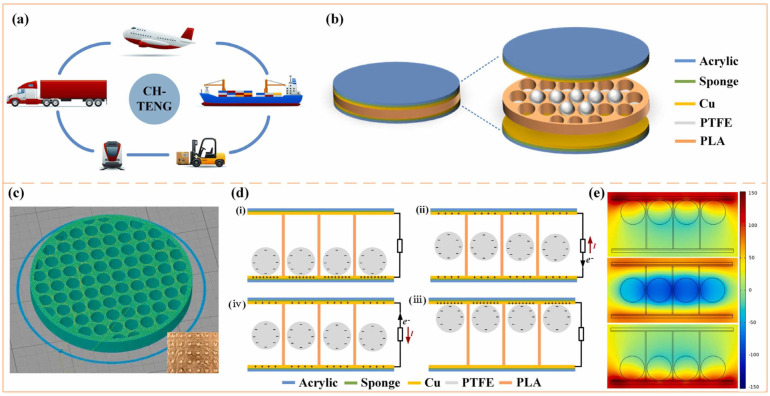
(**a**) Some advanced application scenarios of vibrational energy collecting. (**b**) Advances in friction electric vibration energy harvesters. (**c**) Middle layer architecture. (**d**) Schematic illustration of the energy producing process. (**e**) Potential distribution of CH-TENG obtained by FEM simula-tion [43].

**Figure 9:**
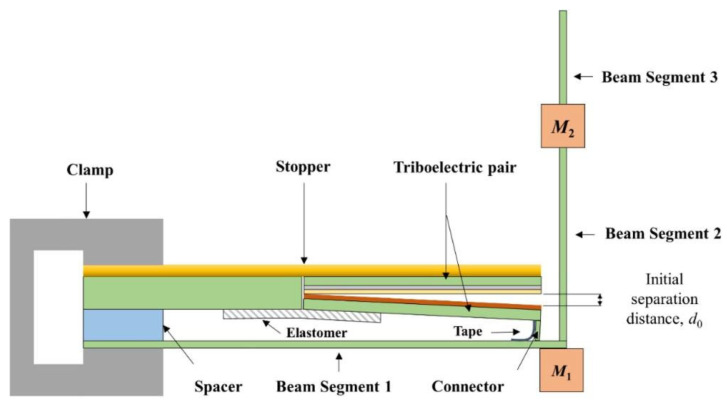
Side view of the structure of LTEH [44].

Friction electric vibration energy harvesters have the advantages of being independent of resonance frequency and high energy conversion efficiency, and as such, they are widely applicable to flexible wearable and self-powered functional sensors, among other applications. Friction electricity is mostly ignored and seldom used in practice because it is very common in life, but at the same time, it is difficult to collect and be utilised. The first practical application of a friction generator was made by academician Wang Zhonglin’s team in 2012, and since then, the friction generator technology started to develop gradually. To address the low output current of friction electric vibration energy harvesters, Yang et al. suggested a scenario using three-dimensionally (3D) integrated multilayer TENGs (Figure 6) [41] with a multilayer structure using acrylic as the support substrate, and eight identical springs were used to connect the movable and fixed pawls. An array of PTFE nanowires was created on the exposed PTFE surface by reactive ion etching using a top-down approach. SEM images of the PTFE nanowires are shown in Figure 6c. The TENG generates enough energy to light over 20 spotlights (0.6 W on average) and a white globe [41]. Qiu et al. in 2020 proposed a friction electric vibration energy collector to collect sound vibration energy, which is based on a porous network structure of copper foam and flexible electrostatically spun polyvinylidene fluoride (PVDF) nanofibres and nylon fabrics in order to construct a novel sandwich-shaped acoustic driver TENG (its structure is shown in Figure 7) [42]. From top to bottom, the first layer consists of copper foam connected by polyvinylidene difluoride (PVDF) nanofibres, while the bottom layer consists of nylon fabric with PEDOT (PSS spinning yarn). Among other things, copper bubbles were used as a capacitor, conductor, and magnifier, and as rubbing and electrically conductive layers for the TENG. Using a lightweight PVDF nanofibre coating and nylon fabric as a vibrating membrane, it can easily produce vibrations at weak sound and collect acoustic energy under broadband frequency. The acoustic TENG has a high friction electrical output, with a max amperage density of 25.01 mA/m^2^, a charging ratio of 20.91 μC/s, a charging conversion rate of 59.85%, and the ability to illuminate 384 LEDs and power an electrochromic device to achieve a reversible colour change. In addition, the acoustic TENG has excellent operational stability [42]. Shi et al. proposed a self-powered circular cellular friction nanogenerator (structure shown in Figure 8) [43], which can be applied to capturing the vibration energy of a synchronous motor. Some application scenarios of CH-TENG are presented in Figure 8a. In Figure 8b, the whole structure is shown on the left edge, and the layered structure is shown on the right edge, where the upper and lower substrates are acrylic sheets with attached foam and copper electrodes, respectively, and the round honeycomb shape in the centre is filled with polytetrafluoroethylene. Figure 8c represents the middle structure of the TENG, i.e., the circular honeycomb structure with several circular holes. Figure 8d represents the whole power generation process. When the PTFE ball makes contact with the copper electrode, the copper electrode acquires a positive charge while the surface of the PTFE ball becomes negatively charged (i) As the ball ascends from its initial position, the separation of these charged surfaces induces free charge carriers to migrate under an external load from the top electrode to the bottom electrode in order to equilibrate the local electric field, thereby generating a transient current (ii) Upon contact between the PTFE ball and the top electrode, all charge carriers are transferred to the bottom electrode, resulting in a positively charged top electrode (iii) Subsequently, as the ball descends from this upper position, charge carriers flow back from the bottom electrode to replenish those at the top electrode, thus producing a reverse current (iv) Figure 8e presents the electrical potential distribution for the FEM simulation. When the synchronous motor achieves the residual rate, the CH-TENG will display visible voltage and output pulses to monitor the condition of the synchronous motor’s resonance [43]. To harvest vibration energy over a wider band of vibration frequencies, Liu et al. put forward a multiple degree frictional electrical energy collector (structure shown in Figure 9) [44], which can collect the vibration power at two operating frequencies. The horizontal segment (segment 1) of the L-shaped beam is clamped at one end and the tip mass (M_1_) at the free end is attached below. One end of the vertical section is attached to the open end of the crossbeam 1, and a verification mass (M_2_) is mounted across the two edges. The open end of profile 1 with M_2_ in the vertical profile is called profile 2, and the rest of the profile is called profile 3. The L-shaped beam configuration is constructed from FR-4 glass-reinforced epoxy resin laminate, which offers excellent elasticity and electrical insulation. Mechanical connectors, 3D printed from ABS plastic, are secured parallel to beam 1 and are separated by acrylic blocks. With a reference excitation speedup of 0.6 g and an outside resistance of 1 MΩ, the TEH achieves a maximum RMS voltage of 9.45 V in the first excitation pattern, 11.56 V in the second excitation mode, and an optimum power of 300 μW with an outside load of 85 MΩ [44]. Yang et al. designed a magneto-liquid triboelectric nanogenerator (ML-TENG) to capture low-frequency vibration power, with a peer-to-peer output voltage of 0.47 V at 7 Hz, a peak output current of 4.57 nA at 8 Hz, and a peak output voltage stabilised at 0.60 V when the vibration amplitude exceeded 9.4 mm [45]. Gao et al. appropriately integrated a suspension and damping structure with a friction-electromagnetic thermoelectric hybrid power generator in a suspension structure, which together realised efficient wind vibration energy collection with damping function [46]. The vibration frequency was 8.3–31.2 Hz, and the wind velocity was 2.9–16.2 m/s, enabling a wide area for both vibration and wind power harvesting. At a resonance rate of 13.6 Hz, the vibration energy collector provides peak V_oc_ and I_sc_ of ±30.5 V and ±1026.6 μA, respectively, with an instant power of 8.2 mW, and is capable of illuminating 250 light-emitting diodes (LEDs) [46]. Zhao et al. proposed a disc-shaped friction power harvester that exploits a mechanism of bistability in the shape of a repulsion pair of motors, and experiments have shown that enhancing the amplitude effectively boosts the output performance. At a rate of 2 Hz and an amperage of 0.06 m, the MAX voltage and power of the device are 9.7 V and 8.6 μW, respectively [47].

Compared with the earlier piezoelectric and electromagnetic vibration energy harvesters, friction electric vibration energy harvesters appeared later, so they are considered an emerging technology, with high output voltage, simple manufacturing, low cost, etc., and at the same time, they also have excellent durability and processability. However, one of the challenges faced by TENG is its relatively low output current, which is a difficult point to overcome in the future. Meanwhile, since friction and vibration exist in every aspect of our lives, friction electric vibration energy harvesters will also be developed towards living in the future and will be utilised in people’s daily lives. Table 4 lists some of the experimental data of the friction electric vibration energy harvester for a more visual comparison.

## 5. Electrostatic Vibration Energy Harvester

### 5.1. The Working Principle of the Electrostatic Vibration Energy Harvester and Its Characteristics

The electrostatic vibration energy gathering approach generates electrical power by varying the amount of capacitance. An introductory voltage needs to be impressed on the capacitor before the vibrational energy gathering construct can begin to deliver electrical energy, and as the vibration of the outside environment leads to changes in the charge stored in the condenser, a flow of charge is created in the circuit, which in turn provides electrical energy to the loads. The advantage of static vibration energy collection methods over other vibration energy collection methods is that they are highly compatible with integrated circuit processes, and it is possible to use relatively mature silicon micromechanical technology to create MEMS variable capacitors, which are more conducive to powering small devices such as wireless sensors.

The capacitance of a capacitor depends on the shape and size of the poles of the conductor and the permittivity of the material insulating the poles; the most common capacitor is the parallel plate capacitor. When the edge effect is neglected, the capacitor capacitance value is

(7) $$C=\varepsilon \frac{s}{d}=\frac{\varepsilon_{r}\varepsilon_{0}S}{d}$$

where S is the effective area of the two parallel plates covering each other (m^2^); d is the distance between the parallel plates (m); ε is the dielectric constant of the medium between the plates (F∙m^−l^); ε_r_ is the relative dielectric constant; ε_0_ = 8.85 × 10^−12^ F/m.

**Figure 10:**
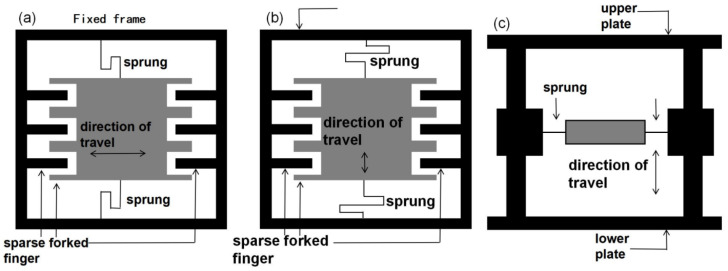
Schematic structure of electrostatic vibration energy harvesting. (**a**) Area-tuned fork-finger variable capacitance. (**b**) Distance-tuned fork-finger variable capacitance. (**c**) Parallel-plate variable capacitance.

According to the different principles of MEMS variable capacitance, electrostatic vibration energy collection systems are classified as parallel-plate variable-capacitance-based vibration energy collection systems, vibration energy harbouring structures built on an area-tuned fork-finger variable capacitor, and vibration energy harbouring architectures built on distance-tuned fork-finger variable capacitance, as shown in Figure 10. The three electrostatic vibratory energy harvesters have different distributions of comb fork fingers as well as different comb fork finger movement directions. According to the capacitance value calculation formula (7) for capacitors, it can be seen that a change in the shape and size of the number of poles will result in a change in the corresponding capacitance value, and each of the three structures has its own advantages according to different scenarios.

### 5.2. Current Research Status of Electrostatic Vibration Energy Harvesters

**Figure 11:**
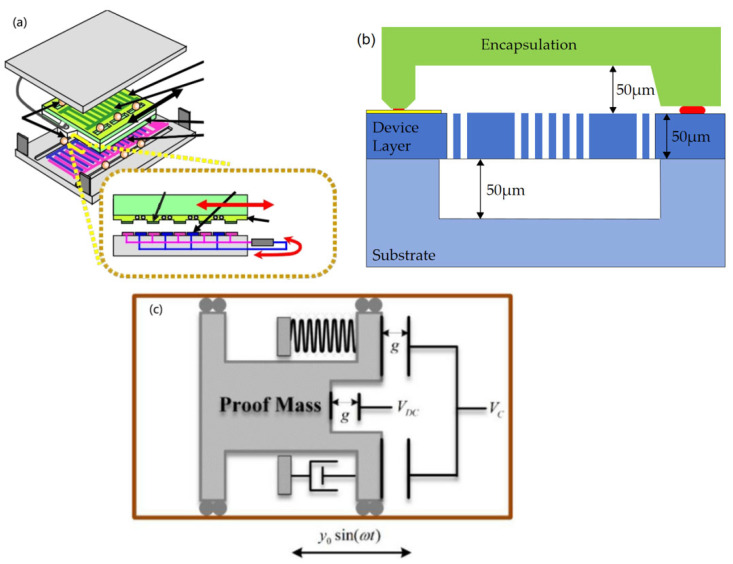
(**a**) New structure of electrostatic microgenerators based on microsphere bearings [48]. (**b**) Cross-sectional view of capacitive energy harvester chip [50]. (**c**) Schematic diagram of an actively tuned capacitive energy harvester [52].

The high energy harvesting efficiency and easy miniaturisation of electrostatic vibration energy harvesters enable better integration with microelectromechanical systems. In 2009, Naruse et al. fabricated an electrostatic microgenerator for low-frequency energy harvesting applications (e.g., Figure 11a) [48]. Separate springs and mass blocks allow for low-frequency remote vibration; separate miniature ball bearings ensure proper clearance maintenance. The generator consists of a fixed part and two silicon subsystems of a novel electrode shape. Microspheres firmly supporting the moving parts are able to follow the deeply grooved rolls among the glass and the silicon baseplate. The generator was able to control the gap between electrodes to achieve a high-power-generating structure with long-distance motion at low frequencies, and could output 40 μW of power at low-vibration frequencies (2 Hz, 0.4 g) [48]. In 2013, Bu et al. [49]. presented a novel electrostatic vibration energy collector featuring polytetrafluoroethylene (PTFE) bulk electrodes, in which a high surface potential can be uniformly obtained on both surfaces by corona charging both sides of the polytetrafluoroethylene (PTFE) bulk electrodes. The results show that the average corona potentials at the higher and the lower faces of the bulk electrolyser are −564.0 V ± 83.1 V and −636.4 V ± 69.7 V at a grid voltage of −700 V. The potential distribution on the two surfaces is homogeneous. The proposed harvester was assembled and measured with sinusoidal vibration terms. The findings showed that the device produces a maximum instantaneous power of 30 μW and a mean power of 5.5 μW at 10 Hz and 2 g peak vibration conditions [49]. In 2014, Kloub et al. proposed a micro-capacitive device for harvesting vibration energy (Figure 11b) [50], which was based on SOI MEMS technology and fabricated in-plane and area-overlapping capacitive energy harvesters on a 6 × 7 mm chip. The chip consists of a 50-micron device layer and an encapsulated wafer, which is bonded with glass frit. The maximum value of capacitance variation is 70pF. Results indicated that the device is capable of producing an export voltage of 5.7 V at an acceleration of 1 g at a bias voltage of 25 V_DC_ [50]. In 2017, Tao et al. [51]. presented a MEMS electret vibration energy collector on a sandwich-based structure, which integrates two oppositely loaded electrets into a singular electrostatic device. The suggested collector is to be able to generate maximum inductance at the highest and lowest terminals, thus improving the output performance. The findings indicate that the export voltages of the proposed sandwich structure e-VEH are enhanced by 80.9% and 18.6% at an elasticity of 5 m/s^2^ against separate top and bottom electret configurations, respectively [51]. Mortaza et al. in 2022 proposed an interactive method for regulating the resonant rate of an electrostatic energy collector (see Figure 11c) [52]. The energy collector is excited by a harmonic basis with value ω and amplitude *y*_0_. As seen in Figure 11c, the sample block is placed on an electret lamp with a V_DC_, and the electret lamp is flanked by two electrodes powered by the outcome of the PID manager. By adding a voltage (V_C_) to these poles, the resonant frequency of the energy collector can be changed due to the softening effect of the electrostatic force, whereby the controller can enhance the efficiency of the power collector by shifting the resonant rate to the left in the frequency domain as long as the stimulus rate is lower than the energy collector’s resonant rate. The findings indicate that the controller can enhance the capacity of the energy harvester by 99.9% in the region below the resonance rate of the energy harvester and by 45.3% in the region above the resonance rate of the energy harvester [52]. In 2020, Ugur et al. presented an energy collector built on push–pull electrostatic transformation and a single charge electret with a size of 4 × 28 cm^3^ for low frequency applications capable of generating voltages in excess of 300 V peak-to-peak and obtaining considerable outputs at frequencies of 5–25 Hz, with 900 μJ of saved energy and 15 μW of electricity [53].

Electrostatic energy harvesters have advantages in terms of low-frequency power generation and integration capability, and they can be used in areas such as automotive tyre pressure monitoring, sensors, and LEDs. However, the application of electrostatic vibrational energy harvesting is somewhat limited by the fact that it requires an external start-up power supply, and that electrostatic energy harvesters generate high voltages, low currents, and high output impedances. Table 5 lists some of the experimental data for electrostatic vibrational energy harvesters for a more visual comparison.

## 6. Magnetostrictive Vibration Energy Harvester

### 6.1. Operating Principle and Characteristics of Magnetostrictive Vibration Energy Harvesters

A magnetostrictive harvester obtains electrical energy from a vibration source by two processes: (1) transformation of physical energy into electromagnetic energy through magnetic interaction with magnetostrictive materials and (2) magnetic power transformed to electrical power by means of an electromagnetic coupler on the circuit. The principle of magnetostriction refers to the fact that when a magnetic material (e.g., iron, nickel, cobalt, and their alloys) is exposed to an applied strong magnetic field, the domains and the lattice of the material itself are altered, resulting in small variations in the shape and dimensions of the whole material. In this case, the fields are deflected in the direction of the magnetic field, while the lattice is stretched or contracted. This small deformation is usually on the nanometre scale but can cause significant deformation when compounding multiple materials. Magnetostrictive materials are classified into two types: single-crystal magnetic materials and polycrystalline magnetic materials. Of these, single-crystal iron-based alloys, for example, are particularly sensitive to changes in magnetic fields. The most widely used are polycrystalline materials, such as iron–nickel alloys and cobalt–iron alloys. The properties of these alloys can be controlled by adjusting the components and processing methods [55].

**Figure 12:**
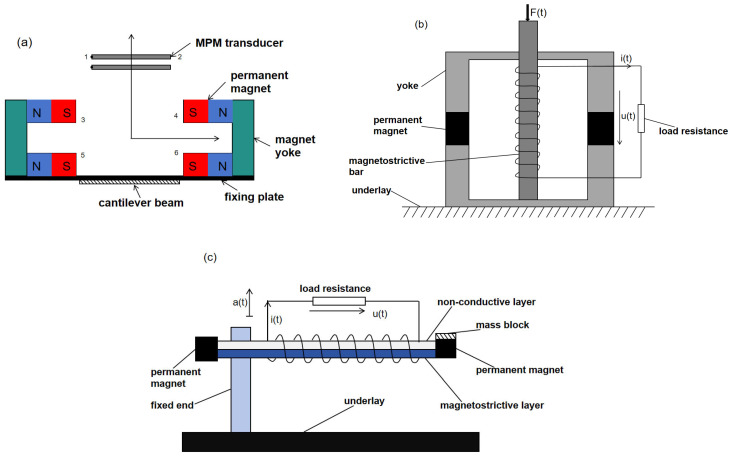
(**a**) Schematic structure of a magnetostrictive vibration energy harvester [56]. (**b**) Direct-drive MVEH [57]. (**c**) Cantilever beam MVEH [57].

The vibratory power harvester consists of four parts: the housing, the magnetic circuit, the cantilever beam, and the magnetoelectric transducer (one structure is shown schematically in Figure 12a) [56]. In the figure, numbers 1 and 2 indicate the left and right sides of Terfenol-D, and numbers 3, 4, 5, and 6 indicate the four magnetic pole faces. The magnetic path consists of four random neodymium–iron–boron motors and two rectangular-shaped yokes. The magnetic circuit is fixed at the front end of the cantilever beam to form a non-uniform mag field in the air gap, which acts as a mass block. The magneto-electric transducer consists of Terfenol-D/PZT/Terfenol-D. The magnetoelectric sensor and the cantilever beam are secured to the casing of the energy collector. When the collector vibrates with the environment, the magnetic loop and the sensor move relative to each other. As the magnetic scene in the air gap is inhomogeneous, the magnetoelectric transducer will sense the varying magnetic field. Under the effect of the varying magnetic area, the magnetostrictive stratum produces mechanical strain and transfers it to the piezoelectric layer to produce electrical energy output and the conversion of mechanical power to electrical power [56].

MVEHs can be broadly classified into two types: one is the direct-drive type, the structure of which is presented in Figure 12b, and the other is the cantilever beam type, wherein the structure is displayed in Figure 12c [57]. In Figure 12b,c, F(t) is the applied driving force, i(t) is the induced current, u(t) is the induced electromotive force, and a(t) is the acceleration vibration excitation. The MVEH mainly consists of magnetostrictive materials, permanent magnets, mass blocks, energy-harvesting coils, and load impedance (or energy harvesting circuit). The magnetostrictive rod or magnetostrictive layer is the core of the generator, the permanent magnet provides the offset magnetic pitch for the magnetic stretching compound, the mass block regulates the generator’s resonant rate, and the coils tightly wound on the magnetostrictive material are connected externally to the load or energy collection circuits for the export of harvested electrical power [57].

### 6.2. Current Status of Research on Magnetostrictive Vibration Energy Harvesters

**Figure 13:**
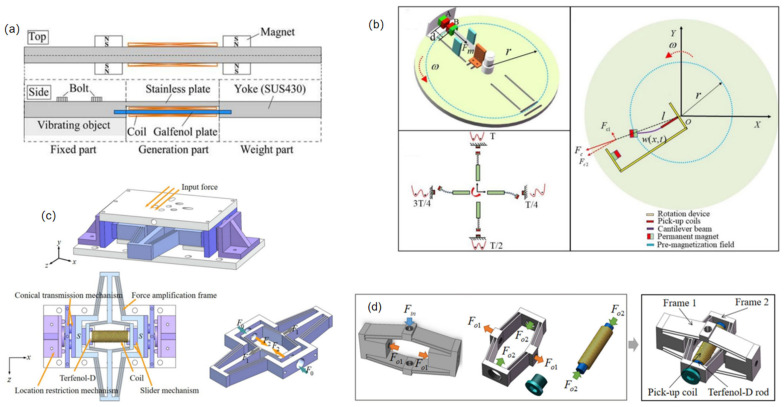
(**a**) Structural diagram of a magnetostrictive vibrational energy harvester made of iron–gallium alloy [58]. (**b**) Schematic diagram of the movement of a bistable magnetostrictive vibration collector in a revolving coordinate system [60]. (**c**) Structural diagram of a magnetostrictive two-dimensional kinetic energy collector [61]. (**d**) Structural diagram of a two-stage rhombus-shaped magnetostrictive vibrational energy harvester device [63].

Magnetostrictive vibration energy harvesters have high mechanical strength to meet the needs of complex machinery and equipment. Magnetostrictive materials, which are the core components of magnetics vibration energy collectors, have been broadly researched by scholars at home and abroad because their strain coefficients and response speeds are larger than those of piezoelectric materials. Shota et al. in 2015 proposed a magnetostrictive vibration energy harvester made of an iron–gallium alloy (Figure 13a) [58], which is built on a parallel-beam architecture and uses a conjugate and Galfenol rods with high resistance to the magnetostrictive effect (stress change of more than 1 T) to efficiently convert vibration into electrical energy. The generator is a cantilever design that includes a generating section made of Galfenol and stainless-steel plates, with coils attached to the Galfenol. The fixing part secures the generator to a vibrating object, while the weight section generates a bending moment in response to inertial forces. Permanent magnets provide bias flux to the Galfenol plate. The generator converts vibrations into electrical energy via the inverse magnetostrictive effect and induction: tensile stress from bending changes the magnetic flux, inducing a voltage across the coil. The results show that it exhibits the highest 35% efficiency at a vibration rate of 202 Hz and an output energy of 4.7 mJ [58]. In the same year, Yan et al. proposed a magnetostrictive-material-based air-gap adjustable vibration energy collector that can be applied to high vibration shock systems, and they structurally designed a capacitive amplifier and a mutable air-gap to improve the energy-collecting effect. The results show that the fabricated magnetostrictive-tech-based vibrational power collector produces higher voltage/watt level voltage and wattage than the conventional intelligent collector and can be successfully used in 20–30 MPa shocks [59]. To increase the working frequency bandwidth and overall collection efficiency of the magnetostrictive vibration power collector, Dong et al. presented a bistable magnetostrictive rotating energy collector incorporating a centrifugal effect in 2024 (e.g., Figure 13b) [60]. The circular disc is a rotating carrier on which a rotating vibration energy collector is mounted. The central frame of the collector consists of a complex cantilever beam cell and a bistable structure. The complex cantilever beam consists of an iron–gallium alloy active layer, a corrugated alloy permeable layer, and an elastomeric one. The complex cantilever beam deflects and deforms during the rotation of the disc. When subject to pressure or tension, the magnetostrictive properties of the material lead to micro-reconfigurations and rotations of the magnetic domains in the crystal structure, which affects the state of magnetisation and changes in magnetic flux density within a substance. Later, together using the Faraday effect of electromagnetic induction, the pick-up coil converts the changing mechanical power into electrical power. The results show to demonstrate that the high energy track oscillator remains stable over the monostable speed band from 230 to 290 rpm. The maximum rms voltage at the collector is 780 mV, which corresponds to a collector power of 4.35 mW [60]. Most of the conventional energy collectors can only collect energy in one direction. Liu et al. in 2024 proposed a magnetostrictive two-phase kinetically active energy collector located below the floor (e.g., Figure 13c) [61], which has the ability to capture power in both vertical and lateral directions and amplify kinetic energy through a two-stage force amplification scheme. Through the cone drive structure, external forces can be directed and converted for energy harvesting in the y or z position. The outer force moves the tapered groove end, which moves in the x-direction under the action of the reaction force. The force is transmitted to the entry S of the magnifying mechanism by a glide fit and is enhanced to Tefeno-D, eliminating the excess force in the y- and z-directions and ensuring the stable transmission of the force. The top plate connects the side plate and limit bodies to keep incoming displacement under control for safety. Terfenol-D rearranges the magnetic domains under axial pressure and biased magnetic field, generating and inducing an electromotive force in the coil, which enables the transformation of mechanical to magnetic and electrical power. The findings show that the peak export power at the collector is 21.2 mW with sinusoidal excitation at an operative rate of 4 Hz. With stochastic stimuli, the maximum output peak voltage is 2.64 V, and the maximum peak power is 170 mW [61]. In order to study the magnetic properties of magnetostrictive materials under different magnetic excitations and compressive stresses, Liu et al. [62]. made a prototype model of an energy harvester with Galfenol and established a nonlinear eigenmodel of Galfenol, and they verified the accuracy of the nonlinear eigenmodel by testing the differences between the prototype model and the nonlinear eigenmodel under different force amplitudes, force frequencies, and load resistances, which showed that the error between the two is less than 4%. The findings indicate that the nonlinear equivalency circuit mode is effective in analysing and forecasting the output voltage characteristics of the MVEH [62]. Liu et al. proposed a two-stage diamond-shaped energy collection device built on Terfenol-D rods, one that is able to gather vibration energy from seats and pressure energy from passengers during bus travelling (Figure 13d) [63]. The device is composed of a force enlargement unit and an energy transformation core unit. The force amplification is achieved using a two-stage diamond-shaped mechanism, comprising two frames: Frame 1 and Frame 2. This amplification system effectively enhances the subtle vibrations from the environment, optimising energy capture while minimising complexity and instability in both the choice of amplification method and the connection between the frames. The export power of the Terfenol-D rods is 1.056 mW with a working rate of 30 Hz and an input pressure of 25.5 N. The power of the device with a random excitation is collected with an open-circuit voltage of 2.92 V and 266 mW, with an import pressure of 50 N [63].

Magnetostrictive materials have high energy density and superior magneto-mechanical coupling properties, being capable of generating significant voltage output when subjected to small magnetic field changes. Compared to piezoelectric transducer ceramics, magnetostrictive materials are capable of generating higher forces at lower voltages, responding faster, and retaining their performance even after being ground into particles. In addition, magnetostrictive elements have the benefits of high export power and wide operating frequency. However, magnetostrictive materials also have a number of drawbacks such as being expensive and having physical size limitations. Table 6 lists some of the experimental data of electrostatic vibratory energy harvesters for a more visual comparison.

## 7. Conclusions

The technology of harvesting vibrational energy from the environment for self-powering is a research priority and hotspot in the area of energy today and in the coming times. Vibration power is widespread in the environment, and the vibration energy collected from the environment can supply energy for micro electronic devices such as micro-nano sensors, thus reducing the use of chemical batteries and pollution of the environment. This article collected some of the operating principles and recent study progress of vibration energy harvesters at home and abroad. The strengths and weaknesses of each method are summarised, and the following conclusions can be drawn:

(1) The electromagnetic vibration energy collector has the virtues of collecting vibration energy with wide bandwidth, large output current, and high output power, but it has the shortcomings of low output voltage, magnetic leakage, and large size.
(2) Piezoelectric vibration energy harvesters have the virtues of simple structure and high energy density, but the piezoelectric material, which is the core component of their composition, suffers from low strength, charging leakage, and easy aging.
(3) Friction electric vibration energy harvesters have the advantages of higher output voltage, simplicity of manufacture, durability, and machinability, but have the drawbacks of low output current and low efficiency in harvesting energy.
(4) Electrostatic vibration energy harvesters have advantages in terms of low-frequency power generation and integration capability, but the need for an additional start-up power supply becomes their fatal flaw, in addition to the disadvantages of low current and high output impedance.
(5) Magnetostrictive vibration energy harvesters have the advantages of high energy density and superior magneto-mechanical coupling coefficients, fast response time, and wide operating frequency, but they also suffer from the shortcomings of large size and high price.

So far, people involved in the development of vibration energy harvesting technology have made greater progress—there have been a variety of vibration energy harvesters one after another, in terms of generators, automobiles, aerospace, and other fields, realising a wide range of uses. However, there are a series of issues, like low energy intensity, low output power, narrow operating rate band, high cost, and production difficulties. In addition, although the capturing of vibrational power from the surroundings for self-powering can be regarded as an infinite source of energy, the actual lifetime of these vibrational energy harvesters is ultimately limited due to factors such as the material lifetime, but their specific lifetimes have not yet been researched. From the study progress of vibration power collecting technology founded on various mechanisms, the following aspects are worthy of in-depth study:

(1) Improve reliability and durability: Optimise the material design of the vibration energy harvester. Piezoelectric, magnetostrictive, and magnetoelectric materials, as the key core components of the vibration energy harvester, are directly related to the strength and service life of the harvester, and the service life of the harvester can be increased and the damage rate can be reduced by designing new materials or by improving the strength and durability of these materials.
(2) Optimise the circuit design: the energy collector includes two parts, energy collection and energy conversion, but in the energy conversion part of the circuit, the energy will be lost, and the way in which to effectively use this part of the energy is a major problem to be faced in the future. Combining with intelligent control technology can be used to achieve intelligent management of the vibration energy collector and optimise the control, to increase the efficiency of energy use, and to reduce the waste of energy.
(3) Design of a composite energy harvester: Most of the existing vibration energy harvesters are in single form, and they seldom combine multiple energy harvesting methods; most of the energy harvesters can only collect energy in a single direction, but the energy in the environment comes from all directions. Research into multi-form, multi-directional vibratory energy collectors can vastly increase the accuracy of power harvesting and can power much more powerful electronic devices. In addition, in order to address the disadvantage of the need for an outside start-up energy source for the electrostatic vibration energy harvester, it is possible to combine the electrostatic type with the piezoelectric and magnetostrictive vibration energy harvesters, thus eliminating the need for an external power supply.
(4) Miniaturise and integrate energy harvesters: current vibrational energy harvesters have large dimensions, making integration difficult. Combined with MEMS technology, smaller-size vibration energy harvesters are designed, which is conducive to the integration of microelectromechanical systems.
(5) Utilisation across multiple purposes and ranges: In addition to mechanical vibration, various movements of the human body produce vibrational energy, such as the heartbeat, pulse beat, and human body walking. The development of vibration energy collectors suitable for different environments and vibration frequencies will enable them to be used in more application scenarios, and in this way, more miniature wearable or portable electronic devices can be derived.

## Figures and Tables

**Table 1:** Experimental results of some electromagnetic-based vibrational energy harvesters.

Authors	Fabric	Frequency (Hz)	Output Voltage/V	Output Power/mW	Power Density/ $(mW\cdot cm^{-3}$)
Cui et al. [13]	Permanent magnets—coils	31	-	3.83	-
Wang et al. [14]	Rack and pinion, bevel gears	-	18.5	2700	-
Peng et al. [15]	Magnets—coils	20	-	37.45	-
Monaco et al. [16]	magnetic levitation solution	4	-	32	-
Sun et al. [17]	Magnets—coils	116	-	27.2	3.6
Lorenzo et al. [18]	Dobby electromagnetic pendulum	9	-	14.4	-
Sun et al. [21]	Spring pendulum	0.85	-	750	-

Note: “-” indicates not mentioned in the literature.

**Table 2:** Some experimental results of a vibrational energy harvester based on the piezoelectric effect.

Piezoelectricity	Electromechanical Coupling Coefficient	Piezoelectric Constant (pC/N)
AlN	0.23	−2.00
CdS	0.26	−5.18
ZnO	0.48	−5.00
BaTiO_3_	0.49	−58.0
PZT-4	0.70	−123
PZT-5H	0.75	−274
LiNbO_3_ (lithium niobate)	0.23	−1.00
PVDF	0.19	21.0

**Table 3:** Partial experimental results based on piezoelectric vibratory energy harvester.

Authors	Fabric	Frequency (Hz)	Output Voltage/V	Load/kΩ	Output Power/μW	Power Density/ $(mW\cdot cm^{-3}$)
Fang et al. [26]	Cantilever beam type	609	0.898	21.4	2.16	-
Shen et al. [27]	Cantilever beam type	461.15	0.16	6	2.15	3.272
Ye et al. [28]	PSN-PZT piezoelectric ceramics	10	52.89	-	35,010	-
Wang et al. [31]	tapered beam	10.06	19.82	-	-	-
Cho et al. [33]	Cantilever beam type	30	-	-	52,500	28.48
Lee et al. [34]	Cantilever beam type	255.9	1.792	150	2.765	-
Remya et al. [35]	Spring-mass block	30	38	2700	-	-
Ramírez et al. [36]	Cantilever beam type	7.91	9.8	1000	96.04	-

Note: “-” indicates not mentioned in the literature.

**Table 4:** Some experimental results based on the friction electric vibration energy harvester.

Authors	Fabric	Frequency (Hz)	Output Voltage/V	Load/MΩ	Short-Circuit Current/ μA	Output Power/mW	Power Density/ $(W\cdot m^{-2}$)	Current Density/ (mA-m^−2^)
Yang et al. [41]	Three-dimensional (3D) integrated multilayer TENGs	-	303	-	-	0.6	104.6	-
Qiu et al. [42]	Sandwich-shaped acoustic drive TENG	125	546.3	-	60.9	-	-	25.01
Shi et al. [43]	circular honeycomb	37	50	-	3.3	-	-	-
Liu et al. [44]	L-shaped beam	-	11.56	85	-	0.3	-	-
Yang et al. [45]	Magnetic fluids	7	0.6	90	0.00457	0.0054	-	-
Gao et al. [46]	Suspension Structure	13.6	30.5		1026.6	8.2	-	-
Zhao et al. [47]	Rejection magnet	2	9.7	-	-	0.0086	-	-

Note: “-” indicates not mentioned in the literature.

**Table 5:** Some experimental results based on the electrostatic vibratory energy harvester.

Authors	Fabric	Frequency (Hz)	Acceleration/(m/s^−2^)	Load/MΩ	Starting Voltage/V	Output Voltage/V	Output Power/ μW
Naruse et al. [48]	Stripe mask electret	2	-	-	-	-	40
Bu et al. [49]	Block electrodes	10	-	-	−700	-	5.5
Kloub et al. [50]	Area overlap	-	1 g	-	25	5.7	-
Tao et al. [51]	Sandwich construction	122.1	5	-	-	-	0.22
Ugur et al. [53]	Electret—variable area	-	-	-	-	300	15
Daisuke et al. [54]	Double electret electret	155	1 g	1	-	-	-

Note: “-” indicates not mentioned in the literature.

**Table 6:** Some experimental results based on the magnetostrictive vibration energy harvester.

Authors	Fabric	Frequency (Hz)	Output Voltage/mV	Output Power/ mW	Power Density/ (mW-cm^−3^)
Shota et al. [58]	Parallel beam construction	202	-	0.73	-
Dong et al. [60]	Cantilever	-	780	4.35	-
Liu et al. [62]	Galfenol rods—excitation coils	-	2.64	170	-
Liu et al. [63]	Double-stage lozenge	30	250	1.056	-
Ueno et al. [64]	Cantilever	212	3000	1.2	3
Carmine et al. [65]	Three Galfenol rods—permanent magnets	100	6	7	

Note: “-” indicates not mentioned in the literature.

## Data Availability

Data will be made available on request.
